# Association between endometriosis and prognosis of ovarian clear cell carcinoma: A systematic review and meta-analysis

**DOI:** 10.12669/pjms.41.10.12816

**Published:** 2025-10

**Authors:** Chaoyou Zhou, Boliang Chu

**Affiliations:** 1 Chaoyou Zhou, Department of Gynecology, Huzhou Maternity & Child Health Care Hospital, Huzhou, Zhejiang Province 313000, P.R. China; 2 Boliang Chu, Department of Gynecology, Huzhou Maternity & Child Health Care Hospital, Huzhou, Zhejiang Province 313000, P.R. China

**Keywords:** Ovarian neoplasms, Mortality, Recurrence, Endometriosis, Prognosis, Meta-analysis

## Abstract

**Objective:**

To examine if endometriosis affected outcomes of ovarian clear cell carcinoma (OCCC).

**Methodology:**

We searched for all study types on the databases of EMBASE, PubMed, Web of Science and Scopus. The search limits were 1^st^ January 2001 to 2^nd^ April 2025. Data on overall survival (OS) and disease-free survival (DFS) was pooled in a random-effect meta-analysis model.

**Results:**

Nineteen studies were included. Pooling of the data showed that endometriosis-associated OCCC had statistically significant improved DFS (HR: 0.80 95% CI: 0.64, 0.99) as compared to non-endometriosis-associated OCCC, but there seemed to be no difference for OS (HR: 0.79 95% CI: 0.62, 1.01) between the two groups. However, the efficacy was not found to be stable on sensitivity analysis and turned significant (for OS) and non-significant (for DFS) on removal of multiple studies. Subgroup analysis based on study location, sample size (>150 or <150), cancer stage (early only or all stages), type of data (crude or adjusted) and follow-up (<5 or >5 years) showed mixed results.

**Conclusions:**

Low-quality evidence from retrospective studies indicates that there may be a tendency of improved DFS in endometriosis-associated OCCC but there may be no difference in OS. Lack of stability of the results on sensitivity and subgroup analyses are important drawbacks that limit strong conclusions.

**Registration No:** PROSPERO: CRD420251021982.

## INTRODUCTION

Endometriosis is a prevalent estrogen-dependent disease affecting 7-15% of women of reproductive age and characterized by the extra-uterine growth of endometrial tissue.^1^ Presentation of the disease can vary from asymptomatic cases to severe pelvic pain, dysmenorrhea and infertility.^2^ Micro-environmental factors like oxidative stress, immune cell dysfunction and inflammation are thought to play a key role in the transformation of ectopic ovarian endometrial cells to invasive malignancies.^3^ Recent meta-analysis shows that endometriosis leads to an almost two-fold increase in risk for ovarian carcinoma, with ovarian clear cell cancer (OCCC) and endometrioid ovarian cancer being the most common.^4^

About 10% of all ovarian carcinomas are OCCCs and their incidence varies from 5% to 25%.^5^ They have distinct biology and are frequently associated with AT-rich interactive domain containing protein 1A (ARID1A) and phosphatidylinositol-4, 5-biphosphate 3-kinase, catalytic subunit α (PIK3CA) mutations and higher expression of hepatocyte nuclear factor-1b, histone deacetylase 6, vascular endothelial growth factor and tissue factor as compared to other histological subtypes.^6^ BRCA1/2 germline mutations are less frequent while deficiency mismatch repair is more common in OCCC.^7^,^8^ The majority of OCCC patients present early and have a favorable clinical outcome, but patients with more advanced disease have a worse prognosis compared to those with high-serous carcinoma.^9^,^10^ These tumors are also found to be chemo resistant with poor response as compared to high grade serous carcinomas.^11^ Given the distinct features of OCCC, it is prudent to study this subtype separately while assessing prognostic factors.

Since a large number of ovarian cancers arise from endometriosis, several studies^12^-^15^ in the past have examined the impact of endometriosis on patient outcomes but with mixed results. A few meta-analysis studies^15^,^16^ have also examined this clinical question, but with a limited number of studies on OCCC. Specifically, only nine studies were included in the last meta-analysis^16^ on OCCC. With the publication of new studies in literature and to provide a more comprehensive and updated evidence with high statistical power, we conducted the present study to examine if there is a difference in patient survival between endometriosis-associated and non-endometriosis-associated OCCC.

## METHODOLOGY

The review was registered on PROSPERO under the number CRD420251021982. The review is presented in accordance with PRISMA guidelines.^17^

### Literature search:

We searched for studies investigating the association between endometriosis and OCCC on the databases of Embase, PubMed, Web of Science and Scopus. The search limits were set from 1^st^ January 2001 to 2^nd^ April 2025. Two reviewers (BC & CZ) conducted the search. Further details of which can be found in Supplementary Table-I.

**Supplementary Table-I:** Search strategy.

PUBMED	
Search	Query
#1	"ovarian neoplasms"[MeSH Terms] OR "ovarian neoplasms"[All Fields] OR "ovarian cancer"[All Fields] OR "ovarian carcinoma"[All Fields] OR "ovary carcinoma"[All Fields] OR "ovary cancer"[All Fields] OR "ovary neoplasm"[All Fields]
#2	"endometriosis"[MeSH Terms] OR "endometriosis"[All Fields] OR "endometrioses"[All Fields]
#3	"prognostic"[All Fields] OR "prognostica*"[All Fields] OR "prognostics"[All Fields] OR "prognosis"[MeSH Terms] OR "prognosis"[All Fields] OR "prognoses"[All Fields] OR "mortality"[MeSH Subheading] OR "mortality"[All Fields] OR "survival"[All Fields] OR "survival"[MeSH Terms] OR "survivability"[All Fields] OR "survivable"[All Fields] OR "survivals"[All Fields] OR "survive"[All Fields] OR "survived"[All Fields] OR "survives"[All Fields] OR "surviving"[All Fields]
#4	#1 AND #2 AND #3
EMBASE	
*Search*	*Query*
#1	(’ovarian carcinoma’/exp OR ’ovarian carcinoma’ OR ’ovarian neoplasms’/exp OR ’ovarian neoplasms’ OR ’ovarian cancer’/exp OR ’ovarian cancer’ OR ’ovary carcinoma’/exp OR ’ovary carcinoma’ OR ’ovary cancer’/exp OR ’ovary cancer’ OR ’ovary neoplasm’/exp OR ’ovary neoplasm’)
#2	(’endometriosis’/exp OR endometriosis OR endometrioses)
#3	(prognostic OR ’prognosis’/exp OR prognosis OR ’survival’/exp OR survival OR ’mortality’/exp OR mortality OR prognostica*)
#4	#1 AND #2 AND #3
WEB OF SCIENCE	
*Search*	*Query*
#1	ALL FIELDS: ((ovarian carcinoma) OR (ovarian neoplasms) OR (ovarian cancer) OR (ovary carcinoma) OR (ovary cancer) OR (ovary neoplasm))
#2	ALL FIELDS: (Endometriosis OR endometrioses)
#3	ALL FIELDS: (prognostic OR prognosis OR survival OR mortality OR prognostica*)
#4	#1 AND #2 AND #3
SCOPUS	
*Search*	*Query*
#1	TITLE-ABS-KEY-AUTH (ovarian neoplasms OR ovarian cancer OR ovarian carcinoma)
#2	TITLE-ABS-KEY-AUTH (endometriosis)
#3	TITLE-ABS-KEY-AUTH (prognostic OR prognosis OR mortality OR survival OR death)
#4	#1 AND #2 AND #3

Initially, duplicate studies were eliminated. The same two reviewers then independently screened the studies for inclusion by assessing abstracts. Relevant studies underwent complete text analysis for final inclusion. Any discrepancies between the two reviewers were resolved by discussion and consensus. Inter-reviewer agreement was measured using kappa statistics. The bibliography of included studies was also manually examined for any other potential articles.

### Inclusion criteria:

- The population of the study was OCCC.
- The exposure variable was the presence of endometriosis which could have been in the same ovary, the opposite ovary, or extraovarian (eOCCC group).
- The population was to be compared with OCCC not associated with endometriosis (non-eOCCC group).
- Outcomes reported were either overall survival (OS) or disease-free survival (DFS).
- Outcomes were presented as crude data or as multiple covariate-adjusted effect sizes.

### Exclusion criteria:

- Studies not specifically on the clear cell variant and reporting combined data of all histological types of ovarian carcinoma.
- Studies not reporting outcome data as effect size with 95% confidence intervals (CI).
- Articles using the same database with overlapping study periods. In such cases, the study with the largest sample size was chosen.
- Non-English language studies.

### Data extraction:

Two reviewers (BC & CZ) conducted the data extraction independently. Data was obtained from the studies regarding the author, publication year, study design, definition of the eOCCC group, cancer stage, use of adjuvant therapy, sample size, age, bilateral cancer, grade of cancer, lymph node metastasis, type of outcome data (adjusted or crude), if adjusted, the variables adjusted and follow-up.

### Risk of bias analysis:

The same two investigators (BC & CZ) applied the Newcastle Ottawa Scale (NOS)^18^ to examine study quality and award a quality score of 0-9 to each article. The assessment was done on three domains, which were selection of study participants, comparability of groups by adjustment for confounding and ascertainment of the exposure or outcome of interest. Disagreements were resolved by discussion and consensus.

### Data analysis:

OS and DFS data were pooled to obtain hazard ratio (HR) and 95% CI. Heterogeneity among studies was assessed through Cochran’s Q statistic and the *I^2^* index. *I^2^* of over 50% and/or *P* < 0.05 indicated significant heterogeneity. We opted for the random-effect meta-analysis model. Sensitivity analysis was used to assess each study’s impact on the pooled outcome. The Egger’s test was used to check for publication bias. Subgroup analysis was conducted based on study location, sample size (>150 or <150), cancer stage included (early only or all stages), type of data (crude or adjusted) and follow-up (<5 or >5 years). For statistical procedures, we used “Comprehensive Meta-analysis” (Version 3).

## RESULTS

**Fig.1:**
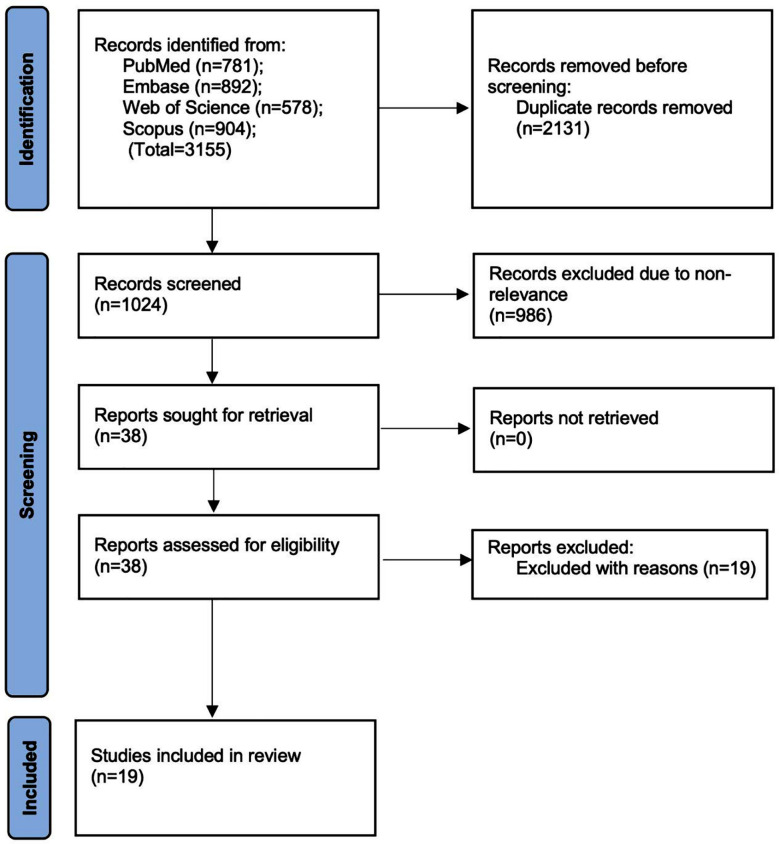
Study flowchart.

The PRISMA flowchart of the study (Fig.1) depicts the screening and selection process of the studies. Nineteen studies^12^-^15^,^19^-^33^ which met the inclusion criteria. The inter-reviewer agreement was high (kappa=0.97).

### Study details:

Baseline information of the studies can be found in Table-I. All were retrospective cohort studies. Endometriosis was confirmed histologically in most studies that reported such data. The definition of eOCCC included the presence of endometriosis in the same ovary, the opposite ovary, or extraovarian tissues. The majority of studies included both early - and advanced-stage OCCC, while two studies included only early-stage disease. Follow-up duration was found to be variable between the included studies.

**Table-I:** Details of included studies.

Study ID Location	Sample size	Age (years)	Bilateral cancer	Lymph node metastasis	Grade III or IV carcinoma	Cancer stage	AC	Outcomes reported	Follow-up
Charatsingha 2021^20^ Thailand	eOCCC: 178, non-eOCCC: 71	eOCCC: 49.7 (arising group), 51.7 (coexisting group); non-eOCCC: 54.3	NR	eOCCC: 10% (coexisting group), 0% (arising group); non-eOCCC: 23.5%	NR	I-IV	Yes	DFS	Up to 12 years
Chou 2022^22^ Taiwan	eOCCC: 209, non-eOCCC: 327	NR	NR	NR	NR	I-IV	Yes	OS, DFS	Mean: 36.6 months
Chung 2025^26^ Korea	eOCCC: 118, non-eOCCC: 141	eOCCC: 45.68, non-eOCCC: 53.95	eOCCC: 66.95%, non-eOCCC: 62.41%	NR	eOCCC: Grade III: 72.03%, Grade IV: 5.08%, non-eOCCC: Grade III: 66.43%, Grade IV: 12.14%	I-IV	Yes	OS	Up to 20 years
Erzen 2001^19^ Slovenia	eOCCC: 6, non-eOCCC: 11	NR	NR	NR	NR	I-IV	NR	OS	Mean 36.6 ± 29.2 months
Gallego 2022^23^ Spain	eOCCC: 33, non-eOCCC: 26	NR	NR	NR	NR	I-IV	Yes	OS	5 years
He 2024^25^ China	eOCCC: 44, non-eOCCC: 61	eOCCC: 45.66, non-eOCCC: 49.05	eOCCC: 11.4%, non-eOCCC: 24.6%	eOCCC: 18.2%, non-eOCCC: 16.4%	NR	I-IV	Yes	OS, DFS	Up to 5 years
Ishibashi 2017^30^ Japan	eOCCC: 45, non-eOCCC: 60	eOCCC: 51, non-eOCCC: 55.5	NR	NR	NR	I-IV	Yes	OS, DFS	Up to 5 years
Katagiri 2012^27^ Japan	eOCCC: 28, non-eOCCC: 32	NR	NR	NR	NR	I-IV	Yes	DFS	Up to 100 months
Kawabata 2017^31^ Japan	eOCCC: 126, non-eOCCC: 42	NR	NR	NR	NR	I	Yes	OS	Median: 91 months
Kim 2015^15^ Korea	eOCCC: 47, non-eOCCC: 62	NR	NR	NR	NR	I-II	Yes	OS, DFS	Mean: 132.2 months for eOCCC and 94.4 for non- eOCCC
Lee 2020^12^ Korea	eOCCC: 107, non-eOCCC: 201	NR	NR	NR	NR	I-IV	Yes	DFS	Median: 31.2 months
Lin 2025^13^ USA	eOCCC: 45, non-eOCCC: 15	NR	NR	NR	NR	I-IV	NR	OS, DFS	Median: 66.7 months
Orezzoli 2008^14^ USA	eOCCC: 41, non-eOCCC: 43	eOCCC: 49, non-eOCCC: 59	NR	NR	NR	I-IV	Yes	OS	Up to 25 years
Park 2018^33^ Korea	eOCCC: 78, non-eOCCC: 77	eOCCC: 48, non-eOCCC: 51	eOCCC: 10.3%, non-eOCCC: 19.5%	eOCCC: 4%, non-eOCCC: 17%	NR	I-IV	Yes	OS, DFS	Median: 71 months
Scarfone 2014^28^ Italy	eOCCC: 27, non-eOCCC: 46	eOCCC: 51.4, non-eOCCC: 58.4	eOCCC: 15%, non-eOCCC: 37%	NR	NR	I-IV	Yes	OS	Median: 68 months
Schnack 2017^32^ Denmark	eOCCC: 80, non-eOCCC: 95	eOCCC: 54, non-eOCCC: 62.9	NR	NR	NR	I-IV	Yes	OS	Up to 10 years
Sun 2023^24^ China	eOCCC: 70, non-eOCCC: 55	eOCCC: 47, non-eOCCC: 57	NR	eOCCC: 5.7%, non-eOCCC: 14.5%	NR	I-IV	Yes	OS, DFS	5 years
Ye 2014^29^ China	eOCCC: 79, non-eOCCC: 131	eOCCC: 46, non-eOCCC: 54	NR	eOCCC: 14.5%, non-eOCCC: 34.3%	NR	I-IV	Yes	OS, DFS	Mean: 51 months
Zhu 2021^21^ China	eOCCC: 16, non-eOCCC: 76	NR	NR	NR	NR	I-IV	Yes	OS, DFS	Mean: 53 months

Endometriosis related ovarian clear cell carcinoma; eOCCC; NR, not reported; AC, Adjuvant chemotherapy; OS, overall survival; DFS, disease-free survival.

Risk of bias analysis conducted by the two reviewers for the included studies is depicted in Supplementary Table-II. Overall, six studies received a perfect score of 9, three studies got a score of 8, nine studies got a score of Seven and one study got a score of Six.

**Supplementary Table-II:** Risk of bias in the included studies.

Study ID	Selection of cohort	Comparability of groups	Outcomes assessment	Final NOS score
Charatsingha 2021^20^	4	-	3	7
Chou 2022^22^	4	2	2	8
Chung 2025^26^	4	-	3	7
Erzen 2001^19^	4	-	2	6
Gallego 2022^23^	4	2	3	9
He 2024^25^	4	-	3	7
Ishibashi 2017^30^	4	2	3	9
Katagiri 2012^27^	4	-	3	7
Kawabata 2017^31^	4	-	3	7
Kim 2015^15^	4	-	3	7
Lee 2020^12^	4	2	2	8
Lin 2025^13^	4	-	3	7
Orezzoli 2008^14^	4	2	3	9
Park 2018^33^	4	2	3	9
Scarfone 2014^28^	4	1	3	8
Schnack 2017^32^	4	2	3	9
Sun 2023^24^	4	-	3	7
Ye 2014^29^	4	2	3	9
Zhu 2021^21^	4	-	3	7

NOS, Newcastle Ottawa Scale.

### Meta-analysis:

**Fig.2:**
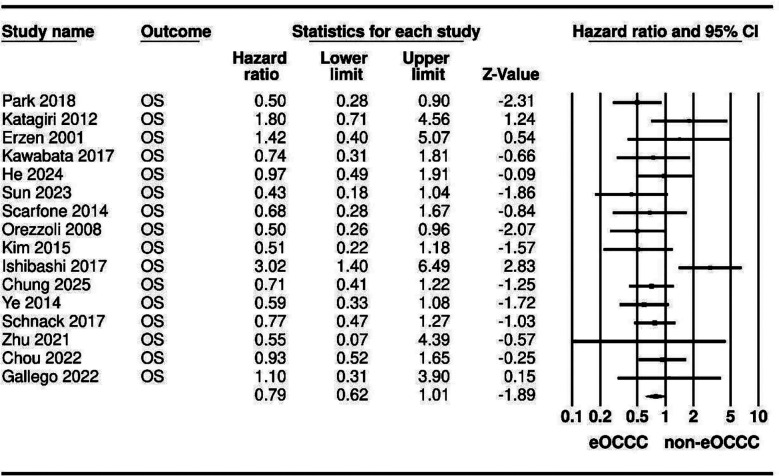
Meta-analysis of the association between endometriosis and OS of OCCC.

Pooling of the data in the meta-analysis showed that endometriosis-associated OCCC had no statistically significant difference for OS as compared to non-endometriosis-associated OCCC (HR: 0.79 95% CI: 0.62, 1.01) (Fig.2). The interstudy heterogeneity was not found to be significant (I^2^=39%). However, the effect size was not found to be stable on sensitivity analysis. Egger’s test was also non-significant (p=0.48).

**Fig.3:**
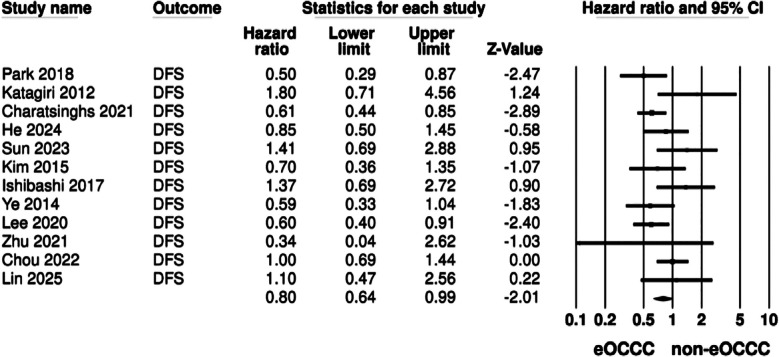
Meta-analysis of the association between endometriosis and DFS of OCCC.

Meta-analysis revealed that endometriosis-associated OCCC had statistically significant improved DFS compared to non-endometriosis-associated OCCC (HR: 0.80 95% CI: 0.64, 0.99) (Fig.3). The interstudy heterogeneity was not found to be significant (I²=41%). Nevertheless, the HR turned non-significant on exclusion of multiple studies Egger’s test was also non-significant (p=0.05). Multiple subgroup analyses were conducted for both OS and DFS (Supplementary Table-III). It can be noted that the HR for OS turned non-significant for all subgroups based on location, cancer stage and data type. Results were found to be significant only for studies with a sample size >150 and follow-up >5 years. For DFS, results were non-significant for all subgroups based on cancer stage, data type and follow-up. However, based on location and sample size, results were significant only for Asian studies and those with a sample size > 150.

**Supplementary Table-III:** Subgroup analysis.

Variable	Groups	Studies	HR (95% CI)	I^2^
***OS***				
Location	America	1	0.5 (0.21, 1.22)	0
	Asia	11	0.81 (0.60, 1.01)	54
	Europe	4	0.86 (0.50, 1.47)	0
Sample size	<150	10	0.90 (0.64, 1.27)	56
	>150	6	0.69 (0.50, 0.97)	0
Stage	All stages	14	0.82 (0.63, 1.06)	45
	Early only	2	0.61 (1.29, 1.27)	0
Data type	Adjusted	8	0.79 (0.58, 1.09)	60
	Crude	8	0.78 (0.53, 1.17)	4
Follow-up	<5 years	3	1.01 (0.59, 1.70)	0
	>5 years	13	0.75 (0.57, 0.98)	47
***DFS***				
Location	America	1	1.10 (0.42, 2.90)	0
	Asia	11	0.79 (0.63, 0.99)	44
Sample size	<150	7	1.05 (0.78, 1.42)	0
	>150	5	0.67 (0.53, 0.83)	38
Stage	All stages	11	0.81 (0.64, 1.03)	46
	Early only	1	0.70 (0.30, 1.60)	0
Data type	Adjusted	5	0.74 (0.54, 1.03)	57
	Crude	7	0.87 (0.63, 1.22)	36
Follow-up	<5 years	9	0.80 (0.54, 1.19)	39
	>5 years	3	0.81 (0.60, 1.07)	47

OS, overall survival; DFS, disease-free survival; HR, hazard ratio; CI, confidence intervals.

## DISCUSSION

The current investigation is the most updated evidence on the impact of endometriosis on survival outcomes of OCCC. We noted that endometriosis-related OCCC was associated with statistically significant improvement in DFS but not in OS as compared to non-endometriosis-related OCCC. The pooled HR showed a 20% improvement in DFS, which was significant, but the 21% difference in the risk of OS was not significant, as the upper end of the 95% CI was just above one. Absence of publication bias and lack of significant heterogeneity in the meta-analysis add to the acceptability of the evidence.

However, lack of stability of the outcomes in sensitivity analysis indicates that the results must be interpreted with caution. Instability of the results could have been due to several factors like methodological heterogeneity between the studies, variations in study populations, cancer stage, treatment protocols, etc. which were further explored in the subgroup analysis. Nevertheless, the lack of stability on sensitivity analysis indicate that the results are not robust and the association between endometriosis-related OCCC and better survival may not be generalized.

The results of our study are partially consistent with the meta-analysis conducted by Chen et al.^16^ In their review, they pooled data from 18 and 12 studies on all ovarian cancer variants to show that endometriosis-associated OCCC had significantly better OS and DFS, respectively. However, their subgroup analysis for OCCC could include a maximum of nine studies (with two pre-2000 studies). While they also noted significantly improved OS and PFS in OCCC related to endometriosis, the number of studies was too low to derive strong conclusions. In this updated review, we excluded very old data and added a large number of contemporary studies to present the most recent evidence on this important topic.

Despite the absence of very high inter-study heterogeneity, there were clear variations between the included studies. The majority of studies were from Asian countries and very few were from Western populations. Subgroup analysis revealed that while the results for the American and European studies were not significant, the results for the Asian studies were either significant or tended to be significant. Literature shows that despite the higher prevalence of ovarian cancer in the West; Asian women have better survival rates. Variations in the type of cancer, diet and lifestyle have been implicated in such differences.^34^

However, one reason for the non-significant results noted in our subgroup analysis could be the scarce data for Western populations. Moreover, methodological heterogeneity between the studies and variations in diagnostic practices and treatment protocols could also have led to such results. Another difference that was noted in the subgroup analysis was the variations in outcomes based on the sample size. Although the reviewers selected a rough number to categorize studies as having a high or low sample size, we observed that articles with a sample size >150 had significant results, whereas those with a sample size <150 had nonsignificant results. This indicates that endometriosis may offer some survival benefit in patients with OCCC and smaller sample-sized studies may have been underpowered to detect significant differences. Likewise, we also noted that studies with longer follow-up (>5 years) found a significant difference in OS in OSCC patients based on endometriosis, indicating that those with shorter follow-up may have missed the difference.

There were also differences among the studies in the type of data reported. About half of the studies in each meta-analysis presented only crude data without taking into account important covariates like age, stage, lymphadenectomy, residual disease, adjuvant therapy, etc. All of these factors have been found to significantly influence outcomes of ovarian carcinoma.^35^-^38^ However, subgroup analysis showed that results were nonsignificant for studies reporting both crude and adjusted data. The only possible reason for this anomaly could be the limited number of studies in each subgroup. Only further studies reporting only adjusted data can resolve the variation in the results.

Endometriosis-related OCCC can be classified into two groups: arising and coexisting. The arising group is characterized by the coexistence of both malignant and benign ectopic endometrial tissues inside the same ovary, suggesting definitive progression from benign endometriosis. The coexisting group is characterized by the presence of both malignant and benign ectopic endometrial tissue in the same patient, without established transition from benign endometriosis. Most of the included studies incorporated both groups in the patient population and did not report separate outcomes. Evidence shows that mutations in ARID1A, PIK3CA and phosphatase and tensin homolog (PTEN) play a role in the transformation of endometriosis to OCCC.^39^,^40^ Such mutations and loss of protein expression are usually found in the arising group but not in the coexisting group.^41^ However, the clinicopathological characteristics are not found to be different between the two groups and there may also be no difference in survival.^15^ Nevertheless, literature is scarce and further investigations comparing the arising and coexisting group are needed.

The tendency of better outcomes in endometriosis-associated OCCC has often been attributed to the earlier diagnosis and younger age of presentation. Patients with OCCC linked to endometriosis may exhibit endometriosis-related symptoms such as pelvic pain, dysmenorrhea and dyspareunia, prompting an earlier consultation with a gynecologist.^26^,^33^ The earlier detection may result in higher possibility of receiving optimal debulking surgery which may improve prognosis.^15^ Another possible explanation for the disparity in prognosis could be the unbalanced patient profiles of the two groups. According to research, patients in the non-endometriosis group were more likely to have unfavorable prognostic markers, such as ascites, a higher CA125 level, advanced stage, ovarian surface invasion, lympho-vascular invasion and omental metastatic disease, than patients in the endometriosis groups.^26^,^27^

However, such results are not reported by all studies. Schnack et al^32^ in their cohort, found that while endometriosis-associated OCCC presented at a younger age, there was no difference in rates of complete cytoreduction at primary debulking surgery or lymphadenectomy between the two groups. There are also differences in molecular characteristics noted between endometriosis-associated OCCC and non-endometriosis-associated OCCC, with the former demonstrating recurrent somatic mutations in PIK3CA and ARID1A.^42^ Research shows that loss of ARID1A expression is a negative prognostic factor in patients with OCCC treated with platinum-based chemotherapy.^27^ However, research on the prognostic role of molecular markers in endometriosis-associated OCCC is still scarce and the improved prognosis of endometriosis-associated OCCC is still attributable to the earlier diagnosis by the majority of studies.^22^,^26^-^28^

### Strengths:

This work presents the most updated evidence on survival in patients with endometriosis-associated OCCC. By excluding very old data and adding a large number of contemporary studies, we have provided significantly improved evidence. A detailed subgroup analysis was conducted which provides better insights to the readers on the effects of different confounders on the overall evidence.

### Limitations:

There are limitations in this meta-analysis. The retrospective design of studies may lead to an increased probability of missing or incomplete data. Moreover, selection bias may also affect the outcomes of retrospective investigations. Missing data in a large number of studies for factors impacting survival, like cancer stage, grade, residual disease, lymphadenectomy status and adjuvant therapy, negatively impacted our capacity to conduct detailed subgroup and meta-regression analyses. Although multivariate analysis was reported in half of the included studies, the inconsistency of covariates used might also have influenced the accuracy of our results. Moreover, there was limited information on prior surgery for endometriosis. A small cohort study has shown that a history of endometriosis surgery may negatively influence OS in endometriosis-associated OCCC.^33^ Lastly, there was a predominance of Asian studies in our review and more data from Western countries is needed to generalize the evidence.

### Clinical implications and future recommendations:

The existence of endometriosis indicates a physiologically unique tumor subtype with a more favorable prognosis, enabling doctors to utilize it as a prognostic indicator during patient consultations. Patients typically exhibit improved responses to conventional surgery and platinum-based chemotherapy, and acknowledging endometriosis-associated OCCC as a possibly less severe variation may assist in preventing overtreatment in particular cases. The enhanced survival rates also indicate specific molecular characteristics, including ARID1A and PIK3CA mutations, alongside less common TP53 alterations, which offer valuable insights for the advancement of targeted therapies and the optimization of personalized management strategies. The correlation between endometriosis and improved outcomes in OCCC facilitates prognostic assessment and treatment planning, while providing reassurance to patients and directing future research towards molecularly customized therapeutics.

Future studies should be of larger sample size matching endometriosis-associated OCCC with non-endometriosis-associated OCCC for age and cancer stage for better evidence. Studies should also report data from Western populations for better generalizability of evidence. Research should also focus on efficacy of targeted therapies based on ARID1A and PIK3CA mutations for endometriosis-associated OCCC.

## CONCLUSIONS

Low-quality evidence from retrospective studies indicates that there may be a tendency of improved DFS in endometriosis-associated OCCC but there may be no difference in OS. Lack of stability of the results on sensitivity and subgroup analyses are important drawbacks that limit strong conclusions.

### Authors’ contributions:

**CZ:** Literature search, Study design and manuscript writing.

**CZ and BC:** Data collection, data analysis and interpretation. Critical review.

**CZ:** Manuscript revision and validation and is responsible for the integrity of the study.

All authors have read and approved the final manuscript.
